# Natural Haemozoin Induces Expression and Release of Human Monocyte Tissue Inhibitor of Metalloproteinase-1

**DOI:** 10.1371/journal.pone.0071468

**Published:** 2013-08-14

**Authors:** Manuela Polimeni, Elena Valente, Daniela Ulliers, Ghislain Opdenakker, Philippe E. Van den Steen, Giuliana Giribaldi, Mauro Prato

**Affiliations:** 1 Dipartimento di Oncologia, Università di Torino, Torino, Italy; 2 Laboratory of Immunobiology, Rega Institute, Catholic University of Leuven, Leuven, Belgium; 3 Dipartimento di Neuroscienze, Università di Torino, Torino, Italy; University of California, Riverside, United States of America

## Abstract

Recently matrix metalloproteinase-9 (MMP-9) and its endogenous inhibitor (tissue inhibitor of metalloproteinase-1, TIMP-1) have been implicated in complicated malaria. *In vivo*, mice with cerebral malaria (CM) display high levels of both MMP-9 and TIMP-1, and in human patients TIMP-1 serum levels directly correlate with disease severity. *In vitro*, natural haemozoin (nHZ, malarial pigment) enhances monocyte MMP-9 expression and release. The present study analyses the effects of nHZ on TIMP-1 regulation in human adherent monocytes. nHZ induced TIMP-1 mRNA expression and protein release, and promoted TNF-α, IL-1β, and MIP-1α/CCL3 production. Blocking antibodies or recombinant cytokines abrogated or mimicked nHZ effects on TIMP-1, respectively. p38 MAPK and NF-κB inhibitors blocked all nHZ effects on TIMP-1 and pro-inflammatory molecules. Still, total gelatinolytic activity was enhanced by nHZ despite TIMP-1 induction. Collectively, these data indicate that nHZ induces inflammation-mediated expression and release of human monocyte TIMP-1 through p38 MAPK- and NF-κB-dependent mechanisms. However, TIMP-1 induction is not sufficient to counterbalance nHZ-dependent MMP-9 enhancement. Future investigation on proteinase-independent functions of TIMP-1 (i.e. cell survival promotion and growth/differentiation inhibition) is needed to clarify the role of TIMP-1 in malaria pathogenesis.

## Introduction

As a consequence of the global eradication program launched by charity foundations [1], World Health Organization (WHO) officially registered in 2010 a decline in estimated malaria cases and deaths, with 655,000 deaths counted among more than 200 million clinical cases worldwide, of which 91% were due to *Plasmodium falciparum* [2]. Nevertheless, malaria remains an alarming emergency in developing countries, with the vast majority of cases occurring in the African Region (81%) and South-East Asia (13%) [2]. Thus it is imperative to investigate new anti-malarial drugs for primary and adjuvant therapy [3] and identify new affordable markers for early diagnosis of malaria.

Human matrix metalloproteinases (MMPs) are a family of proteolytic enzymes involved in wide variety of biological functions including modulation of inflammatory response, disruption of inter-endothelial tight junctions, and degradation of sub-endothelial basal lamina [4]–[7]. As such, they are good candidate molecules and indeed there is growing evidence that MMPs play critical roles in malaria in both animal and human disease models (see [8]–[10] for more extensive reviews). Notably, malarial pigment (nHZ, natural haemozoin), a waste product of haemoglobin digestion by *Plasmodium* parasites, induces MMP-9 release from human monocytes [11]–[14] and endothelial cells [9], [15]–[16], and synthetic HZ (sHZ) interacts with proMMP-9 priming its activation by MMP-3 [17].

Endogenous inhibitors of MMPs (TIMPs, tissue inhibitors of metalloproteinases) represent one mode of MMP regulation [18]; however, their involvement in malaria has been scarcely investigated, and their role remains debated. A few lines of evidence from animal and human models support the involvement of TIMPs in malaria. CM-sensitive mice infected with *P. berghei ANKA* display increased mRNA expression of TIMP-1 in the brain, and both TIMP-1 and -3 mRNA is increased in the liver and spleen, whilst mRNA levels of TIMP-2 and -4 remain unchanged [19]. Increased serum levels of TIMP-1 are also found in Rhesus macaques (*Macaca mulatta*) experimentally infected with *P. coatneyi*, a simian malaria parasite that closely mimics the biological characteristics of *P. falciparum* and replicates the multisystemic dysfunction of human severe malaria [20]. Human patients with severe or uncomplicated malaria have higher serum TIMP-1 levels compared to healthy controls suggesting TIMP-1 may be a valuable marker of disease severity [21]. However, the cellular source of TIMP-1 and the mechanisms underlying TIMP-1 enhancement are as of yet unidentified. Additionally, it is imperative to assess whether increased CM-associated TIMP-1 levels are sufficient to counterbalance nHZ-enhanced MMP-9.

Endothelial cells and monocytes are both producers of inducible TIMP-1 protein [22]–[23], and their phenotype and functions can be directly affected by malarial products such as infected red blood cells (IRBCs) and nHZ [9]. However, it is unlikely that endothelium is the TIMP-1 source in malaria, as neither IRBCs nor nHZ affect TIMP-1 protein release from human microvascular endothelial cells [15]–[16].

Here we investigate the *in vitro* effects of nHZ on human monocyte TIMP-1 gene expression and protein release, the responsible soluble mediators, the signaling pathways involved, and the net gelatinolytic activity resulting from the altered MMP-9/TIMP-1 balance.

## Materials and Methods

### Materials

All materials were from Sigma-Aldrich, St Louis, MO, unless otherwise stated. Sterile plastics were from Costar, Cambridge, UK; Percoll was from Pharmacia, Uppsala, Sweden; Diff-Quik parasite stain was from Baxter Dade AG, Dudingen, Switzerland; Panserin 601 monocyte medium was from PAN Biotech, Aidenbach, Germany; ELISA kits for hTNF-α and hIL-1β assays were from Cayman, Ann Arbor, MI; blocking anti-hTNF-α/IL-1β antibodies and rhTNF-α/IL-1β were from Merck, Darmstadt, Germany; ELISA kit for hMIP-1α/CCL3, anti-hMIP-1α/CCL3 blocking antibodies and rhMIP-1α/CCL3 were from R&D Systems, Minneapolis, MN; p38 MAPK inhibitor SB203580 was from Cell Signaling Technology, Danvers, MA; ELISA kits for hMMP-9 and hTIMP-1 were from RayBiotech, Norcross, GA; cell culture medium RPMI, DQ-gelatin, TRIzol, M-MLV, oligo-dT, sense and anti-sense primers, Platinum Taq DNA Polymerase were from Invitrogen, Carlsbad, CA; DNA-free kit was from Ambion, Austin, TX; Beacon Designer 7.0 software was from Premier Biosoft International, Palo Alto, CA; dNTPs were from Applied Biosystem, Foster City, CA; anti-hTIMP-1 (sc-21734) monoclonal antibodies were from Santa Cruz Biotechnology, Santa Cruz, CA; iCycler iQ Real Time Detection System Software version 3.0 and electrophoresis reagents were from Bio-Rad Laboratories, Hercules, CA; computerized densitometer Chemidoc was from Biorad, Hercules, CA; Synergy HT microplate reader was from Bio-Tek Instruments, Winooski, VT; recombinant proMMP-9 and MMP-9 were produced as previously described [24].

### Cultivation of *Plasmodium falciparum* and Isolation of nHZ

*Plasmodium falciparum* parasites (Palo Alto strain, Mycoplasma-free, LPS-free) were kept in culture as described [25]. After centrifugation at 5,000*g* on a discontinuous Percoll-mannitol density gradient, nHZ was collected from the 0–40% interphase. nHZ was washed five times with 10 mM HEPES (pH 8.0) containing 10 mM mannitol at 4°C and once with phosphate-buffered saline (PBS). nHZ was treated with DNase to remove any adhering nuclear material as previously described [26]. nHZ was stored at 20% (v/v) in PBS at −20°C or immediately used for opsonization and phagocytosis.

### Preparation and Handling of Monocytes

Human monocytes were separated by Ficoll centrifugation [11] from freshly collected buffy coats discarded from blood donations by healthy adult donors of both sexes provided by the local blood bank (AVIS, Associazione Volontari Italiani Sangue, Torino, Italy). Separated lympho/monocytes were resuspended in RPMI medium and plated on six-well plates. Each well received 8×10^6^ cells. The plates were incubated in a humidified CO_2/_air-incubator at 37°C for 60 min. Thereafter, non-adherent cells were removed by three washes with RPMI and remaining adherent cells (∼1×10^6^ monocytes/well) were again incubated at 37°C overnight. Shortly before starting phagocytosis, wells were washed with RPMI and Panserin 601 monocyte medium was added (2 ml/well).

### Pre-selection of NF-κB-non-activated Monocytes by FACS Analysis and Real Time RT-PCR

Before starting experiments, a pre-selection of cell populations was taken as a precautionary measure, as previously described [13]. Briefly, cell cultures isolated through Ficoll separation were analyzed by flow cytometry. Only cell populations showing at least 70% monocytes were used for following experiments. Additionally, in order to avoid the use of NF-κB pre-activated monocytes, cells were analyzed by Real Time RT-PCR: in each cell preparation a cell aliquot was stimulated or not with LPS (1 µg/ml) for 4 h, and TNF-α RNA production measured in lysates by Real Time RT-PCR. Glyceraldehyde-3-phosphate dehydrogenase (GAPDH) was used as housekeeping gene. Only unstimulated monocyte populations (NF-κB-non-activated cells) showing at least a 3-PCR-cycles gap of cDNA amplification between controls and LPS-stimulated cells were used for the subsequent experiments.

### Cell Culturing Experimental Conditions: Phagocytosis of nHZ or Latex Particles and Treatment with Blocking Antibodies, Recombinant Pro-inflammatory Molecules, or Cell Signalling Chemical Inhibitors

Phagocytosis assay was performed as previously described [27]. Briefly, nHZ (120 nmoles HZ haem, an amount comparable to 50 µl trophozoites on haem content basis) and 50 µl amine-modified latex particles (2.5% solids, diameter 0.105 µm) were added to each well of a six-well plate containing the same amounts of human adherent monocytes (∼1×10^6^ cells/well). nHZ and latex particles were opsonized with fresh autologous serum. After opsonization, all phagocytic meals were suspended in Panserin 601 monocyte medium. The plates were centrifuged at low speed for 5 s to start phagocytosis and incubated in a humidified CO_2_/air-incubator at 37°C for 2 h - a time period maximizing phagocytosis but not sufficient to induce haem-oxygenase-mediated degradation of ingested haem [28]. Cells were checked by optical microscopy: as an average, nHZ-containing monocytes were 25–35% among the total cells, a percentage similar to *in vivo* levels measured in patients with severe malaria showing high parasitaemia [29]. Additionally, the amount of nHZ phagocytosed by monocytes was quantified by luminescence: as an average, each monocyte ingested nHZ equivalent to ∼8–10 trophozoites in term of ingested haem, in line with previous results from our group [30]. Thereafter, non-ingested nHZ and latex particles were removed by four washes with RPMI. The plates were then incubated in Panserin 601 medium in a humidified CO_2_/air-incubator at 37°C for the indicated times.

In selected experiments, unfed and nHZ-fed monocytes were incubated for the indicated times with 30 ng/ml anti-hTNF-α, anti-hIL-1β or anti-hMIP-1α/CCL3 blocking antibodies; 20 ng/ml rhTNF-α, rhIL-1β or rhMIP-1α/CCL3; 10 µM SB203580, 15 µM quercetin, 10 µM artemisinin, or 10 µM parthenolide, dissolved in DMSO (final solvent concentration less than 0.1% v/v).

Complimentary co-culturing experiments with unfed and nHZ-fed monocytes were also performed using six-well plate Transwell systems with 0.4 µm of porosity (see Figure S2A-B). Briefly, monocytes (0,5×10^6^ cells/transwell) were seeded in transwells (provisionally placed in cell-free wells) and fed with nHZ for 2h. Thereafter, transwells with nHZ-fed monocytes were moved into new wells containing unfed monocytes (1×10^6^ cells/well) and incubated for 2 h, a time previously shown to be sufficient to induce early production of TNF-α, IL-1β, MIP-1α/CCL3 from nHZ-fed cells [25], [31]. As negative and positive controls, respectively, unfed monocytes were also left unfed or fed with nHZ for 2 h. Then nHZ and transwells with nHZ-fed monocytes were removed. After washing and adding fresh medium, cells were incubated in Panserin 601 medium in a humidified CO_2_/air-incubator for 24 h before collecting supernatants for subsequent analyses.

### Cytotoxicity Studies by Lactate Dehydrogenase Assay

The potential cytotoxic effects of phagocytic meals (nHZ and latex) and treatments (anti-hTNF-α, anti-hIL-1β and anti-hMIP-1α/CCL3 antibodies; rhTNF-α, rhIL-1β and rhMIP-1α/CCL3; SB203580, quercetin, artemisinin, and parthenolide) were measured as the release of lactate dehydrogenase (LDH) from cells into the extracellular medium (see Figure S1A–B).

Briefly, human adherent monocytes were left unfed or fed with phagocytic meals for 2 h; after washings, unfed and fed cells were incubated for 24 h in the presence or in the absence of each one of the above mentioned treatments. Then, 1 ml of cell supernatants was collected and centrifuged at 13000 *g* for 2 min. Cells were washed with fresh medium, detached with trypsin/ethylenediaminetetraacetic acid (EDTA) (0.05/0.02% v/v), washed with PBS, resuspended in 1 ml of TRAP (82.3 mM triethanolamine, pH 7.6), and sonicated on ice with a 10 s burst. 5 µl of cell lysates and 50 µl of cell supernatants were diluted with TRAP and supplemented with 0.5 mM sodium pyruvate and 0.25 mM NADH (300 µL as a final volume) to start the reaction. The reaction was followed measuring the absorbance at 340 nm (37°C) with a microplate reader. Both intracellular and extracellular enzyme activities were expressed as µmol of oxidized NADH/min/well. Finally, cytotoxicity was calculated as the net ratio between extracellular and total (intracellular+extracellular) LDH activities. Cell viability was calculated as the net ratio between intracellular and total LDH activities.

### Measurement of TIMP-1 mRNA Levels by Real Time RT-PCR

After the end of phagocytosis, monocytes (1×10^6^ cells/well) were further incubated with Panserin 601 monocyte medium in a humidified CO_2/_air-incubator at 37°C for 15 h. Total cellular RNA from 2×10^6^ cells (contained in 2 wells) was isolated from monocytes by TRIzol, according to the manufacturer’s instructions, and eluted in 20 µl diethyl pyrocarbonate water. To remove any contaminating DNA, RNA was treated with Ambion’s DNA-free kit. Subsequently, 6 µg of RNA were reverse transcribed into single-stranded cDNA using M-MLV (200 U/µl final concentration) and oligo-dT (25 µg/µl final concentration). Real Time RT-PCR analysis was performed with the i*Cycler* Instrument and the iCycler iQ Real Time Detection System Software version 3.0 as previously described [11]. For TIMP-1 (GenBank accession no. BC000866) primers (forward: 5′-AGA CGG CCT TCT GCA ATT CC-3′, reverse: 5′-GCT GGT ATA AGG TGG TCT GGT T-3′), oligonucleotide sequences were identified using Beacon Designer Software package and designed to be intron-spanning allowing the differentiation between cDNA and genomic DNA-derived PCR products. GAPDH (GenBank accession no. BC020308) was used as housekeeping gene; primer sequences were from the Bio-Rad library: forward: 5′-GAA GGT GAA GGT CGG AGT-3′ and reverse: 5′-CAT GGG TGG AAT CAT ATT GGA A-3′. For each 25 µl PCR reaction mix: 1 µl cDNA (corresponding to 10^5^ cells); 1.0 µl sense primer (10 µM); 1.0 µl anti-sense primer (10 µM); 0.5 µl dNTP (10 mM); 1.5 µl MgCl_2_ (50 mM); 1.25 U Platinum Taq DNA Polymerase; 2.5 µl Buffer (10x); 1.7 µl SYBR Green (stock 1∶10,000); and 14.55 µl PCR-grade water were mixed together. DNA polymerase was pre-activated for 2 min at 94°C. TIMP-1 amplification was performed by 50 cycles with denaturation at 54°C for 30 s, annealing at 57°C for 40 s and extension at 72°C for 30 s; GAPDH amplification was performed by 40 cycles with denaturation at 94°C for 30 s, annealing at 65°C for 30 s and extension at 72°C for 30 s for GAPDH. Relative quantification for TIMP-1, expressed as fold variation over untreated control cells, was calculated with the efficiency-corrected quantification model [32] after determining the difference between C_T_ of the given gene A (TIMP-1) and that of the calibrator gene B (GAPDH). C_T_ values are means of triplicate measurements. To validate the use of the method we tested serial dilutions of cDNA from monocytes, stimulated for 15 h by 20 ng/ml rhTNF-α. The specificity of PCR was confirmed by melt curve analysis. The melting temperatures for each amplification product were 87.4°C for TIMP-1 and 86.5°C for GAPDH.

### Assay of TIMP-1 Protein Levels by Western Blotting

After the end of phagocytosis, monocytes (1×10^6^ cells/well) were further incubated with Panserin 601 monocyte medium in a humidified CO_2/_air-incubator at 37°C for 24 h. Thereafter, 15 µl of cell supernatants were separated on a 12% polyacrylamide denaturing gel, transferred to a polyvinylidene difluoride membrane, and probed with monoclonal anti-TIMP-1 antibodies. After staining with secondary anti-mouse HRP-conjugated antibodies, bands were visualized by ECL staining. Densitometric analysis of the bands considered to reflect total protein levels was performed using a computerized densitometer with protein levels presented in relative units compared to background.

### Measurement of TNF-α, IL-1β, MIP-1α/CCL3, MMP-9 and TIMP-1 Production by ELISA

After the end of phagocytosis, monocytes (1×10^6^ cells/well) were further incubated with Panserin 601 monocyte medium in a humidified CO_2/_air-incubator at 37°C up to 24 h. Cell supernatants were collected 6 h, 15 h and 24 h after phagocytosis. The levels of soluble TNF-α, IL-1β, MIP-1α/CCL3, MMP-9 and TIMP-1 were assayed in 100 µl of monocyte supernatants by specific ELISA. Standard calibration curves were generated with rhTNF, rhIL-1β, rhMIP-1α/CCL3, rhMMP-9 and rhTIMP-1 according to the manufacturer’s instructions.

### Assay of Total Gelatinolytic Activity by a Fluorogenic Gelatin Conversion Assay

After phagocytosis, monocytes (1×10^6^ cells/well) were further incubated with Panserin 601 monocyte medium in a humidified CO_2/_air-incubator at 37°C for 24 h. Thereafter, the total gelatinolytic activity [the net overall activity of gelatinases (MMP-2 and MMP-9) and their inhibitors (TIMP-2 and TIMP-1, respectively)], was measured in cultured supernatants using DQ-gelatin as recently described [33]. Briefly, DQ-gelatin was dissolved at a stock concentration of 1 mg/ml in pure water. Serial dilutions of the samples were prepared in a black microtiter plate, and DQ-gelatin was added at a final dilution of 2.5 µg/ml in 50 mM Tris/HCl pH 7.6, 150 mM NaCl, 5 mM CaCl_2_, 0.01% Tween-20. Immediately thereafter, the plate was placed in the fluorescence reader and fluorescence measured every 10 min for 2 h at 37°C (ex. 485 nm/em. 530 nm). As a standard, serial dilutions of recombinant MMP-9 activated by MMP-3 were included in each plate. The results were expressed as gelatinolytic activity units per ml, with 1 unit corresponding to 37 ng/ml activated rhMMP-9.

### Statistical Analysis

For each set of experiments, data are shown as means+SEM (Real Time RT-PCR, densitometry, ELISA and fluorogenic gelatin conversion assay) or as a representative image (Western blotting) of three independent experiments with similar results. All data were analyzed by a one-way Analysis of Variance (ANOVA) followed by Tukey’s post-hoc test (software: SPSS 16.0 for Windows, SPSS Inc., Chicago, IL).

## Results

### nHZ Induces TIMP-1 mRNA Expression and Protein Release in Human Adherent Monocytes

**Figure 1:**
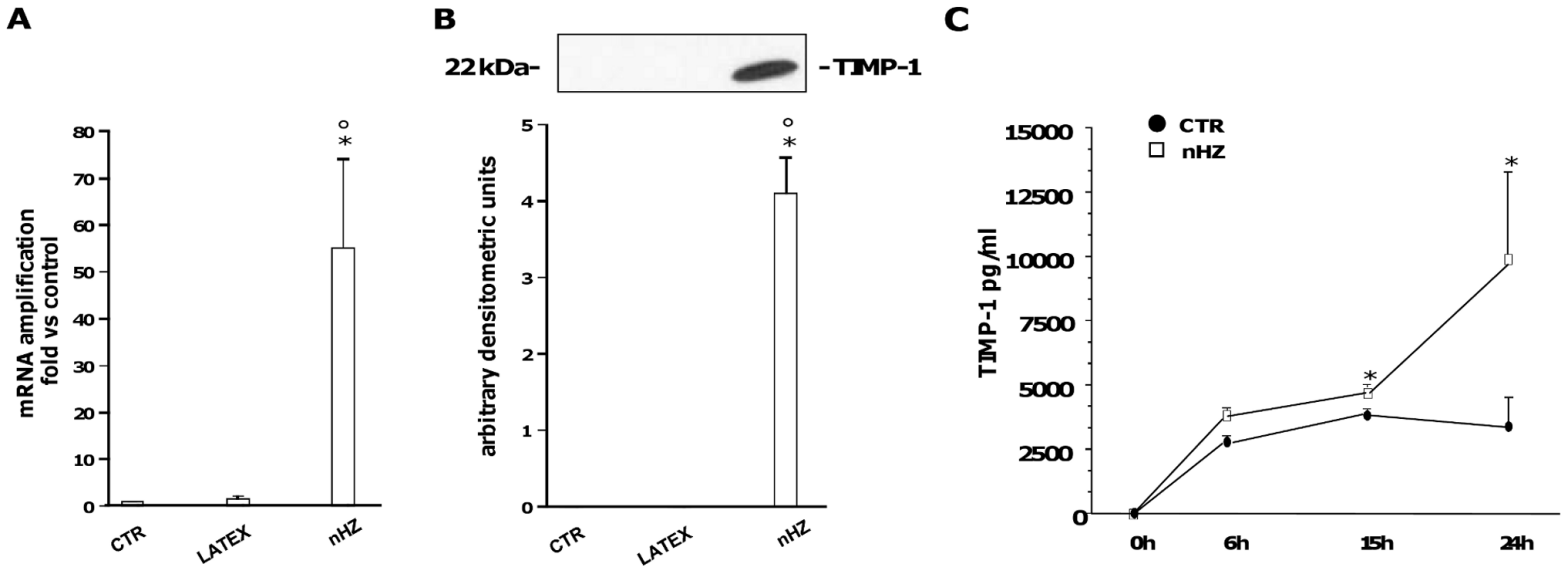
nHZ induces TIMP-1 mRNA expression and protein release in human adherent monocytes. Cells were left unfed (CTR), fed with latex, or fed with nHZ for 2 h. Subsequently, monocytes were washed and incubated for 15 h, and TIMP-1 mRNA levels were measured in cell lysates by Real Time RT-PCR (Panel A); alternatively, monocytes were washed and incubated for 24 h, and TIMP-1 protein levels were evaluated in 24 h cell supernatants by Western blotting and densitometry (Panel B) or in 6, 15, and 24 h cell supernatants by ELISA (Panel C). Data are indicated as mean values+SEM or as a representative blot of three independent experiments. All data were evaluated for significance by ANOVA. Panel A: Vs CTR cells (column 1) *p<0.05; Vs LATEX-fed cells (column 2) °p<0.05. Panel B: Vs CTR cells (column 1) *p<0.0001; Vs LATEX-fed cells (column 2) °p<0.0001. Panel C: Vs CTR cells (black circled line) *p<0.0001.

Human adherent monocytes (1×10^6^/well) were left unfed (control cells, CTR), fed with latex as a control meal (LATEX-FED), or fed with nHZ (nHZ-FED) for 2 h. At the end of phagocytosis cells were washed and incubated for 15 h. Thereafter, mRNA was extracted and TIMP-1 expression was measured by Real Time RT-PCR (Figure 1A). nHZ enhanced >50-fold TIMP-1 mRNA expression compared to CTR, while latex particles did not significantly alter basal TIMP-1 mRNA levels. Alternatively, to evaluate TIMP-1 protein secretion, monocytes were washed and incubated for 24 h after phagocytosis, with cell supernatants collected at selected time-points (0, 6, 15, and 24 h) during the incubation. TIMP-1 protein levels were evaluated by Western blotting and densitometry (24 h end-point studies, Figure 1B) and by ELISA (0–24 h time course studies, Figure 1C). TIMP-1 protein release was induced *de novo* in nHZ-FED cells, whereas Western blotting analysis did not detect apparent levels of TIMP-1 in 24 h supernatants of CTR and LATEX-FED cells. To verify these results, we used a more sensitive ELISA assay to examine levels of TIMP-1 at earlier time-points. Over time, CTR cells released low levels of TIMP-1 protein (up to ∼3,5 ng/ml at the end of the 24 h observational period), which were significantly increased upon nHZ treatment in the nHZ-FED cells at 15 h and 24 h post phagocytosis (up to ∼10,5 ng/ml at the end of the 24 h observational period). Notably, nHZ and latex did not display any cytotoxic effects on monocytes, and did not affect cell viability (see Figure S1A–B).

We next questioned whether nHZ-FED monocytes could modulate TIMP-1 secretion from unfed cells. To address this possibility, we co-cultured both unfed and nHZ-fed monocytes in six-well plate Transwell systems with 0.4 µm of porosity, plating unfed monocytes (1×10^6^ cells/well) at the bottom of the wells and seeding nHZ-fed human adherent monocytes (0,5×10^6^ cells/well) onto the inserts. Co-cultures were incubated for 2 h before removal of the inserts. After washing, unfed monocytes were further incubated for 24 h. Then, cell supernatants were collected and analysed for TIMP-1 secretion. Results (see Figure S2A) show that unfed monocytes co-cultured with nHZ-fed monocytes secrete higher amounts of TIMP-1 than control cells, albeit significantly lower in comparison to the amounts released by nHZ-fed cells cultured alone.

### Role of TNF-α, IL-1β, and MIP-1α/CCL3 in nHZ-dependent TIMP-1 Induction

**Figure 2:**
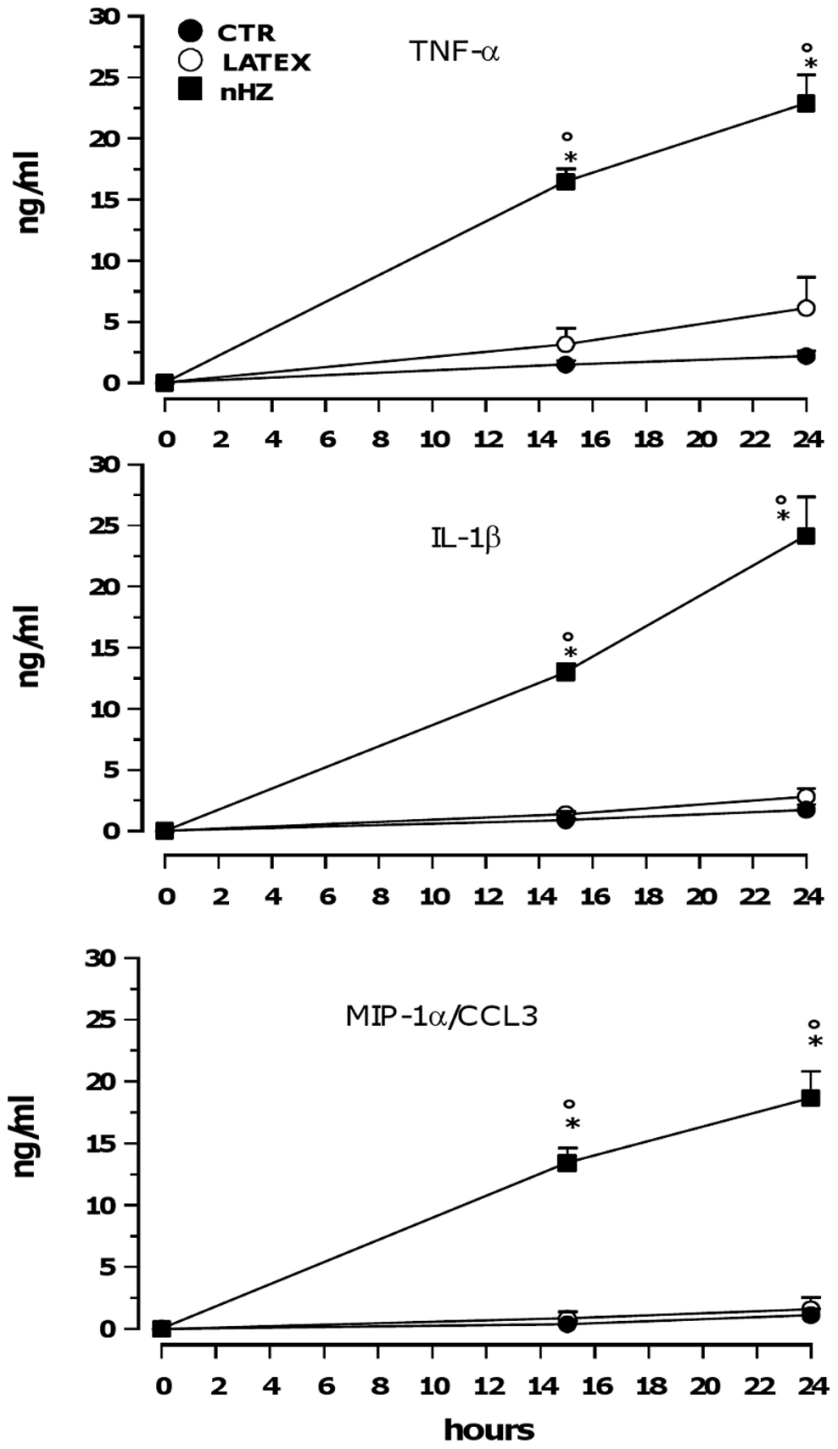
nHZ enhances production of TNF-α, IL-1β, and MIP-1α/CCL3 by human adherent monocytes. Cells were left unfed (CTR), fed with latex particles (LATEX), or fed with nHZ (nHZ) for 2 h; therefore, production of TNF-α (upper panel), IL-1β (central panel), and MIP-1α/CCL3 (lower panel) was monitored in cell supernatants 0, 15 and 24 h after the end of phagocytosis. Data are means+SEM of three independent experiments. Production of pro-inflammatory molecules is indicated as ng/ml. All data were evaluated for significance by ANOVA. Vs control cells (CTR) *p<0.0001; Vs LATEX-fed cells (LATEX) °p<0.0001.

Protein levels of TNF-α, IL-1β, and MIP-1α/CCL3 released from CTR, LATEX-FED and nHZ-FED monocytes (1×10^6^/well) were measured over 24 h post phagocytosis by ELISA (Figure 2). During the observational period, CTR and LATEX-FED cells released low amounts of all three pro-inflammatory molecules in a time-dependent manner, reaching less than 5 ng/ml TNF-α and less than 3 ng/ml IL-1β or MIP-1α/CCL3 at 24 h as evaluated for both conditions. No significant differences between CTR and LATEX-FED cells were found. After phagocytosis of nHZ, the production of pro-inflammatory molecules was significantly higher than in CTR or LATEX-FED cells, reaching ∼15 ng/ml TNF-α and ∼12 ng/ml IL-1β and MIP-1α/CCL3 at 15 h, as well as ∼22 ng/ml TNF-α or IL-1β and ∼15 ng/ml MIP-1α/CCL3 at 24 h.

**Figure 3:**
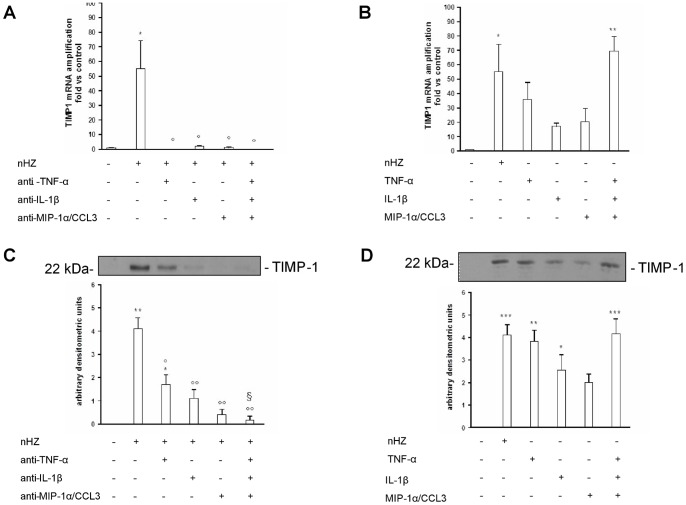
Role of TNF-α, IL-1β, and MIP-1α/CCL3 in nHZ-induced mRNA expression and protein release of human monocytic TIMP-1. Human adherent monocytes were left unfed (negative controls) or fed with nHZ (positive controls). Thereafter, cells were incubated for 15 h (mRNA studies, Panels A-B) or 24 h (protein studies, Panels C-D) in the presence/absence of the following treatments: single or combined doses (30 ng/ml) of anti-hTNF-α, anti-hIL-1β and anti-hMIP-1α/CCL3 blocking antibodies (Panels A and C, blocking approach); and single or combined doses (20 ng/ml) of rhTNF-α, rhIL-1β and rhMIP-1α/CCL3 (Panels B and D, mimicking approach). After lysis, TIMP-1 mRNA expression was measured by Real Time RT-PCR; alternatively, TIMP-1 protein levels in cell supernatants were evaluated by Western blotting and densitometry. Data are indicated as mean values+SEM or as a representative blot of three independent experiments. All data were evaluated for significance by ANOVA. Panel A: Vs unstimulated cells (column 1) *p<0.01; Vs untreated nHZ-fed cells (column 2) °p<0.05. Panel B: Vs unstimulated cells (column 1) *p<0.05, **p<0.01. Panel C: Vs unstimulated cells (column 1) *p<0.05, **p<0.0001; Vs untreated nHZ-fed cells (column 2) °p<0.01, °°p<0.0001; Vs anti-TNFα-treated nHZ-fed cells (column 3) §p<0.05. Panel D: Vs unstimulated cells (column 1) *p<0.05, **p<0.01, ***p<0.001.

As a next step, a double blocking and mimicking approach was used to investigate the role of IL-1β, TNF-α, and MIP-1α/CCL3 (alone or combined) in the nHZ-dependent induction of TIMP-1 mRNA expression and protein release (Figure 3).

After the end of phagocytosis CTR and nHZ-FED cells (1×10^6^/well) were left untreated or treated with single or combined doses (30 ng/ml) of anti-hTNF-α, anti-hIL-1β and anti-hMIP-1α/CCL3 blocking antibodies for 15–24 h. We analyzed TIMP-1 mRNA expression and protein release by Real Time RT-PCR in cell lysates (15 h) and Western blotting in cell supernatants (24 h), respectively. The effects of nHZ on either mRNA expression or protein release were reduced by single blocking antibodies, and were fully abrogated by the combination of all three antibodies (Panels 3A and 3C). Neither basal TIMP-1 mRNA nor TIMP-1 protein levels in CTR cells were affected by any treatments with blocking antibodies and by non-immune Ig used as a negative control (data not shown).

Alternatively, after the end of phagocytosis CTR and nHZ-FED cells were left untreated or treated with single or combined doses (20 ng/ml) of rhTNF-α, rhIL-1β and rhMIP-1α/CCL3. Again, TIMP-1 mRNA expression and protein release were analyzed as previously indicated. The effects of nHZ on either mRNA expression or protein release were only partially mimicked by adding the single recombinant molecules to CTR cells, whereas they were fully recapitulated by the simultaneous addition of all three recombinant molecules (Panels 3B and 3D). None of treatments with recombinant molecules further induced TIMP-1 mRNA or protein levels in nHZ-FED cells, possibly because a plateau was already reached after nHZ phagocytosis (data not shown).

Of note, blocking antibodies and recombinant molecules did not display any cytotoxic effects on monocytes and did not affect cell viability (see Figure S1A-B).

### Involvement of p38 MAPK Pathway in nHZ-induced Release of TIMP-1 and Pro-inflammatory Molecules

**Figure 4:**
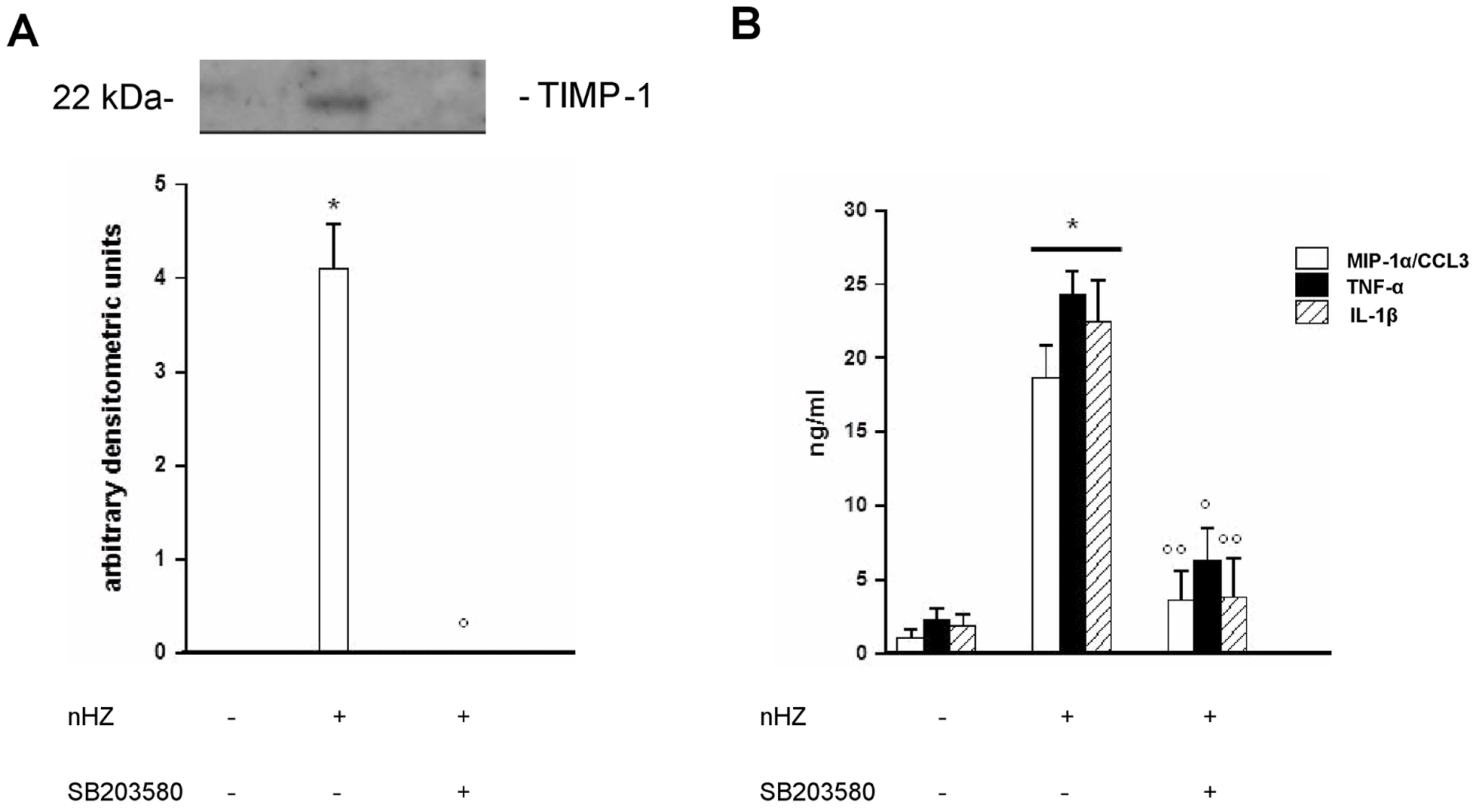
Involvement of p38 MAPK pathway in nHZ-induced release of TIMP-1 and related pro-inflammatory molecules from human adherent monocytes. Cells were left unfed (negative controls) or fed with nHZ (positive controls) for 2 h; after phagocytosis, cells were incubated for 24 h alone or with 10 µM SB203580 (p38 MAPK synthetic inhibitor). Thereafter, cell supernatants were collected. TIMP-1 protein release was analyzed by Western blotting and densitometry (Panel A); TNF-α, IL-1β, and MIP-1α/CCL3 production was measured by ELISA (Panel B). Data are indicated as mean values+SEM or as a representative blot of three independent experiments. All data were evaluated for significance by ANOVA. Panel A: Vs unstimulated cells (column 1) *p<0.0001; Vs untreated nHZ-fed cells (column 2) °p<0.0001. Panel B: Vs unstimulated cells (dataset 1) *p<0.0001; Vs untreated nHZ-fed cells (dataset 2) °p<0.01, °°p<0.0001.

Human adherent monocytes (1×10^6^/well) were left unfed or fed with nHZ for 2 h and then incubated for an additional 24 h in the presence or absence of p38 MAPK inhibitor SB203580 (10 µM) [34]. Supernatants were collected and TIMP-1 release was evaluated by Western blotting and subsequent densitometry, whereas TNF-α, IL-1β, and MIP-1α/CCL3 production was measured by ELISA (Figure 4, panel A: TIMP-1 protein release; panel B: production of pro-inflammatory molecules).

Addition of SB203580 abrogated nHZ-induced protein release of TIMP-1, TNF-α, IL-1β, and MIP-1α/CCL3. SB203580 treatment did not affect basal protein levels of TIMP-1 or pro-inflammatory molecules in CTR cell supernatants (data not shown). SB203580 did not display any cytotoxic effects on monocytes and did not affect cell viability (see Figure S1A–B).

### Involvement of NF-κB Pathway in nHZ-induced Release of TIMP-1 and Pro-inflammatory Molecules

**Figure 5:**
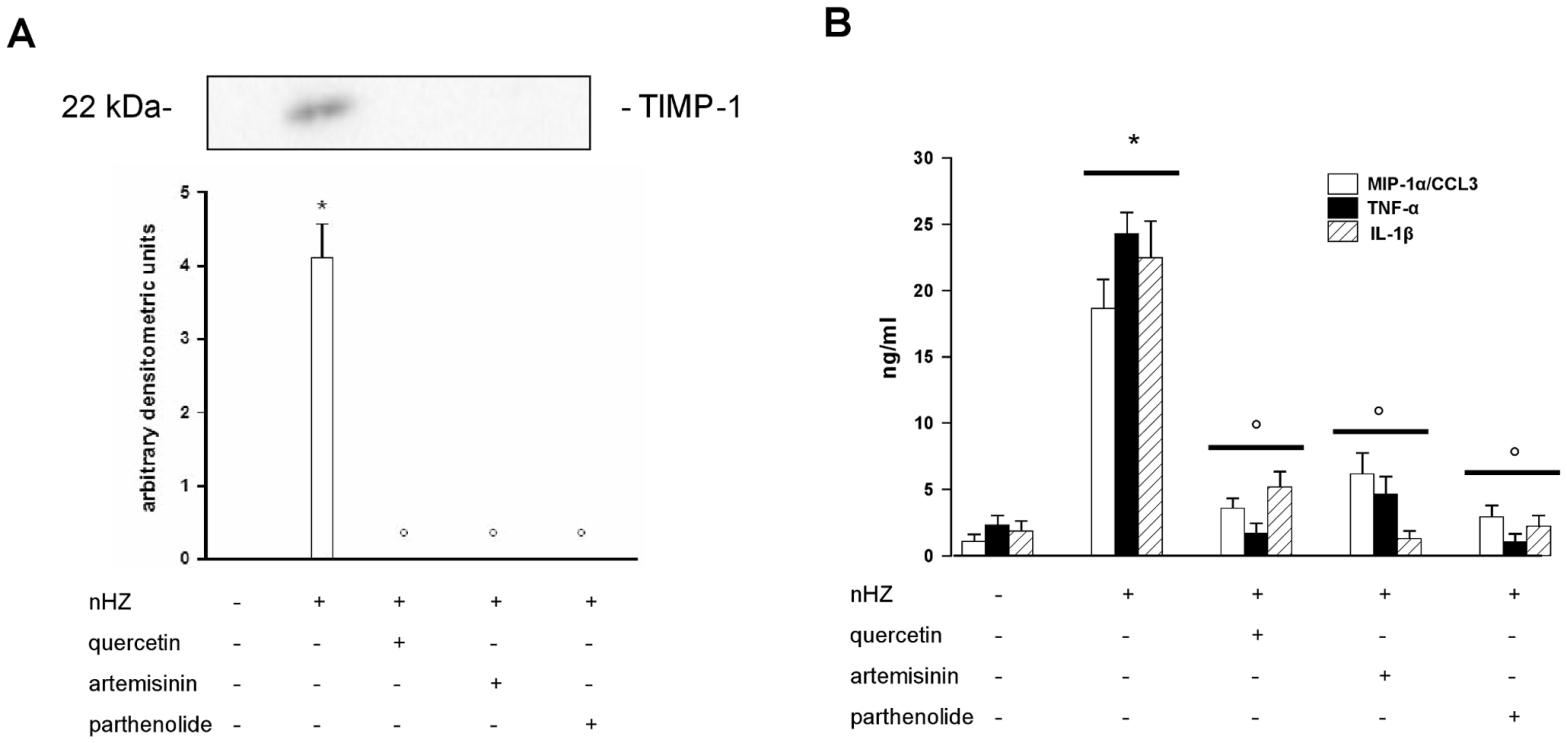
Involvement of NF-κB pathway in nHZ-induced release of TIMP-1 and related pro-inflammatory molecules from human adherent monocytes. Cells were left unfed (negative controls) or fed with nHZ (positive controls) for 2 h. After phagocytosis, cells were incubated for 24 h alone or with 15 µM quercetin, 10 µM artemisinin and 10 µM parthenolide (NF-κB inhibitors). Thereafter, cell supernatants were collected. TIMP-1 protein release was analyzed by Western blotting and densitometry (Panel A); TNF-α, IL-1β, and MIP-1α/CCL3 production was measured by ELISA (Panel B). Data are indicated as mean values+SEM or as a representative blot of three independent experiments. All data were evaluated for significance by ANOVA. Panel A: Vs unstimulated cells (column 1) *p<0.0001; Vs untreated nHZ-fed cells (column 2) ° p<0.0001. Panel B: Vs unstimulated cells (dataset 1) *p<0.0001; Vs untreated nHZ-fed cells (dataset 2) ° p<0.0001.

Human adherent monocytes (1×10^6^/well) were left unfed or fed with nHZ for 2 h and then incubated for additional 24 h in the presence or absence of three inhibitory molecules which have been reported to block NF-κB signaling at different levels: 15 µM quercetin, inhibitor of I-κBα phosphorylation and subsequent degradation [35]; 10 µM artemisinin, inhibitor of NF-κB nuclear translocation [36]; and 10 µM parthenolide, inhibitor of NF-κB binding to DNA [37]. After treatment with the denoted inhibitors, cell supernatants were collected and TIMP-1 release was evaluated by Western blotting and subsequent densitometry, while TNF-α, IL-1β, and MIP-1α/CCL3 production was measured by ELISA (Figure 5, panel A: TIMP-1 protein release; panel B: pro-inflammatory molecules production).

nHZ-induced protein release of TIMP-1, TNF-α, IL-1β, and MIP-1α/CCL3 was abrogated by all three NF-κB inhibitors. Quercetin, artemisinin, and parthenolide did not affect basal protein levels of TIMP-1 or pro-inflammatory molecules in CTR cell supernatants (data not shown). NF-κB inhibitors did not display any cytotoxic effects on monocytes, and did not affect cell viability (see Figure S1A–B).

### nHZ Enhances Total Gelatinolytic Activity of Human Monocytes Despite TIMP-1 Induction

Unfed and nHZ-fed monocytes (1×10^6^/well) were incubated for 24 h in the presence or absence of p38 MAPK or NF-κB inhibitors. After collection of cell supernatants 6, 15, and 24 h after phagocytosis, MMP-9 and TIMP-1 protein levels were quantified by ELISA, and stoichiometric ratios were calculated. Both MMP-9 and TIMP-1 levels were higher in nHZ-fed cell supernatants compared to unfed cells at all time-points. However, MMP-9/TIMP-1 stoichiometric ratios were >1 both in unfed and nHZ-fed cell supernatants (with the exception of unfed cells at 6 h of the observational period), suggesting that nHZ-dependent TIMP-1 induction is not sufficient to counteract nHZ-dependent MMP-9 increase (panels 6A–B).

**Figure 6:**
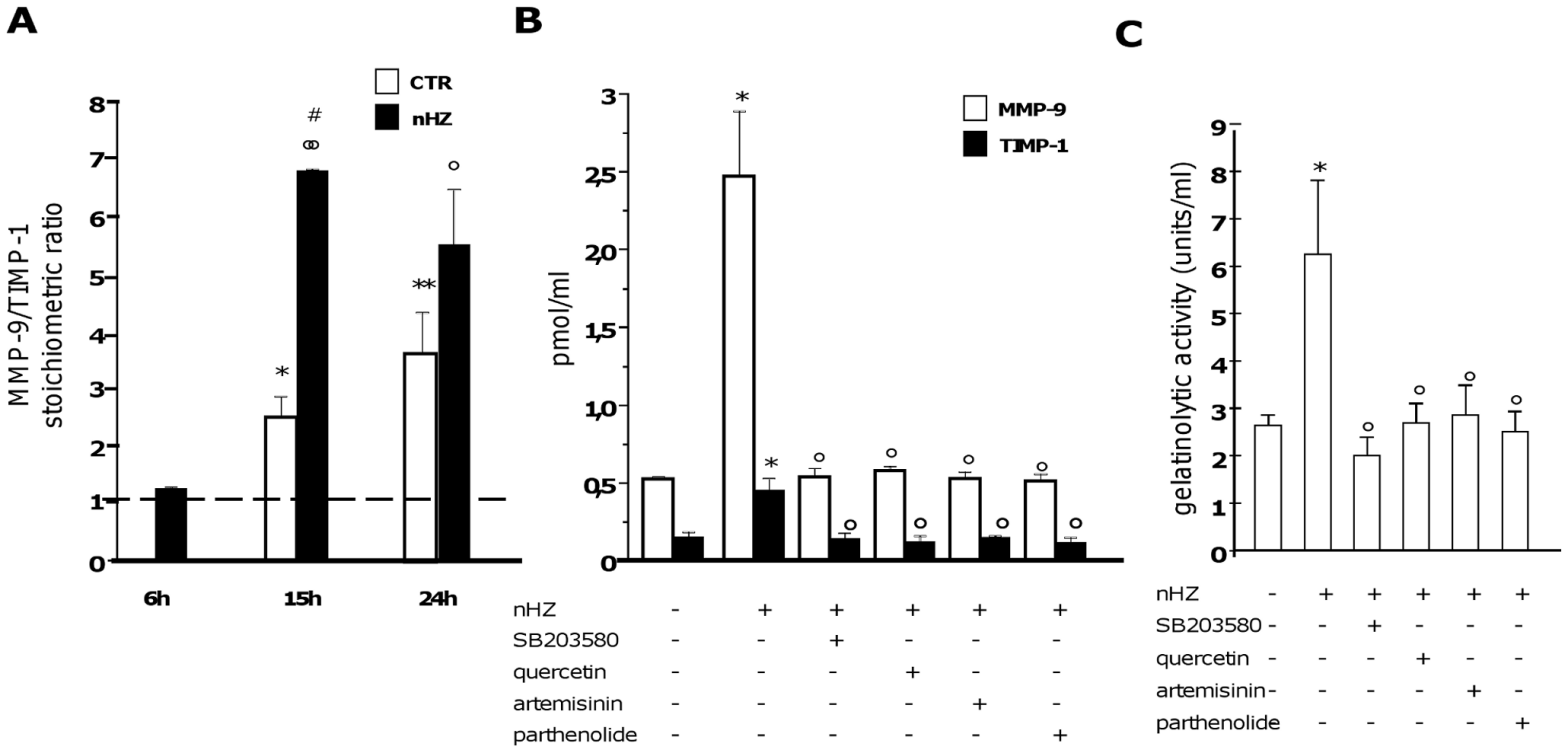
nHZ-dependent induction of TIMP-1 secretion does not counterbalance nHZ-dependent MMP-9 enhancement in human adherent monocytes. Cells were left unfed or fed with nHZ for 2 h; after washing, cells were incubated for 24 h alone or with 10 µM SB203580 (p38 MAPK synthetic inhibitor), 15 µM quercetin, 10 µM artemisinin and 10 µM parthenolide (NF-κB inhibitors). During the incubation, cell supernatants were collected at different time-points (6, 15 and 24 h). Thereafter, MMP-9 and TIMP-1 protein levels were measured by ELISA, and MMP-9/TIMP-1 ratios were calculated; additionally, total gelatinolytic activity was analyzed by fluorogenic gelatin conversion assay. Panel A. MMP-9/TIMP-1 stoichiometric ratios in unfed (white columns) and nHZ-fed (black columns) cell supernatants during time (6, 15 and 24 after the end of phagocytosis). Panel B. MMP-9 (white columns) and TIMP-1 (black columns) protein levels (expressed as pmol/ml) in unfed and nHZ-fed cell supernatants, in the presence/absence of p38 MAPK and NF-κB inhibitors. Panel C. Gelatinolytic activity (expressed as gelatinolytic activity units per ml, with one gelatinolytic unit corresponding to 37 ng activated rhMMP-9) in unfed and nHZ-fed cell supernatants, in the presence/absence of p38 MAPK and NF-κB inhibitors. Data are mean values+SEM of three independent experiments. All data were evaluated for significance by ANOVA. Panel A: Vs unfed cells (6 h) *p<0.05, **p<0.005; Vs nHZ-fed cells (6 h) °p<0.001, °°p<0.0001; Vs unfed cells (15 h) #p<0.0001. Panel B: Vs unstimulated cells (column 1) *p<0.0001; Vs untreated nHZ-fed cells (column 2) °p<0.0001. Panel C: Vs unstimulated cells (column 1) *p<0.05; Vs untreated nHZ-fed cells (column 2) °p<0.05.

We next sought to determine any overall net changes in gelatinolytic activity by employing a fluorogenic gelatin conversion assay [33] (Figure 6C). Consistently with data on MMP-9/TIMP-1 stoichiometric ratios obtained by ELISA, total gelatinolytic activity was enhanced after nHZ phagocytosis. As expected, p38 MAPK inhibitor and NF-κB inhibitors abrogated all nHZ effects (panels 6B–C).

It is interesting to speculate if nHZ-fed monocytes can modulate TIMP-1 and MMP-9 secretion of unfed cells. We tested this possibility by co-culturing both unfed and nHZ-fed monocytes in six-well plate Transwell systems and assessing changes in TIMP-1 and MMP-9 protein levels. Results show that unfed monocytes co-cultured with nHZ-fed monocytes secrete higher amounts of TIMP-1 and MMP-9 compared to controls, although the levels are significantly lower compared to nHZ-fed cells alone (see Figure S2B). Nevertheless, in all cases MMP-9/TIMP-1 ratios were >1, confirming that nHZ-dependent TIMP-1 induction is not sufficient to counteract nHZ-dependent MMP-9 increase.

## Discussion

TIMP molecules are widely distributed in the animal kingdom, with four paralogous genes encoding TIMP-1 to -4 in the human genome [18]. TIMPs were originally characterized as endogenous MMP inhibitors due to their ability to bind zinc in the MMP active site and thereby antagonize the effects of activated/cleaved MMPs and block enzyme activity. TIMPs are key regulators of crucial MMP-related pathophysiological processes, such as turnover of extracellular matrix and shedding of cell surface molecules [38]. Additionally, TIMPs have important roles in a broad spectrum of biological activities often independent of MMPs, including effects on cell survival, cell growth and differentiation, cell migration, angiogenesis, and synaptic plasticity [39]–[40].

It has only recently come to light that TIMPs are involved in malaria disease state. Currently, our understanding of TIMPs in malaria manifests from only a few studies showing dysregulated TIMP levels in different *in vivo* and *in vitro* malaria models. *In vivo*, brain and spleen TIMP-1 mRNA levels are increased in CM-sensitive mice infected with *P. berghei ANKA* [19]. Further, increased TIMP-1 serum levels are associated with severe malaria in Rhesus macaques infected with *P. coatneyi* [20]. Moreover, in a case-control study on Gabonese children, TIMP-1 serum levels were significantly higher in malaria patients than in healthy controls and correlated with disease severity, suggesting a potential role for TIMP-1 as a diagnostic marker [21]. *In vitro*, *P. falciparum*-IRBCs and nHZ promoted TIMP-2 but not TIMP-1 protein release from human microvascular endothelial cells [15]–[16]. On the other hand, in human monocytes, nHZ did not affect TIMP-2 production [41], whereas no data on TIMP-1 regulation by nHZ were available so far.

The present work explores the effects of phagocytosed nHZ on monocyte TIMP-1 expression and release, and identifies cellular mechanisms and soluble mediators involved. Untreated cells, used as negative controls, express low TIMP-1 mRNA levels and secrete negligible TIMP-1 protein, consistent with previous reports [23], [42]. Interestingly, 15 h after phagocytosis of nHZ, TIMP-1 mRNA expression is significantly enhanced, and higher TIMP-1 protein levels are detected in cell supernatants up to 24 h post nHZ phagocytosis. The effect is specific for nHZ and not due to phagocytosis *per se* since latex particles do not induce TIMP-1 expression and secretion.

These data give insight into the molecular mechanisms that enable nHZ to alter the phenotype of human monocytes. Indeed, nHZ was previously shown to induce gene expression of a large number of pro-inflammatory molecules including cytokines (IL-1β, TNF-α, IL-1RA) and chemokines (MIP-1α/CCL3, MIP-1β/CCL4, MCP-1/CCL2, IL-8/CXCL8, ENA-78/CXCL5, GROα/CXCL1, GROβ/CXCL2, GROγ/CXCL3) [43]. Also, nHZ was reported to promote expression and release of MMP-9 [11]–[13] and lysozyme [24], [31]. Notably, it has been proposed that IL-1β, TNF-α and MIP-1α/CCL3 function as soluble mediators for nHZ-dependent upregulation of either MMP-9 [11]–[12], [14] or lysozyme [24], [31]. Here we show that these three pro-inflammatory molecules are also involved in nHZ-induced expression and release of TIMP-1. Either 15 or 24 h after phagocytosis of nHZ, the levels of TNF-α, IL-1β, and MIP-1α/CCL3 in cell supernatants are significantly higher compared to controls. Treating with blocking antibodies directed against these three molecules abrogates the effects of nHZ on monocyte TIMP-1 mRNA expression and protein release. Consistently, unfed cells treated with recombinant TNF-α, IL-1β, and MIP-1α/CCL3 display TIMP-1 mRNA levels in lysates and TIMP-1 protein levels in supernatants similar to those of nHZ-fed cells. These results are in line with previous data showing TIMP-1 induction by IL-1β [44]. Apparently, all three molecules are required in combination to obtain nHZ-dependent TIMP-1 induction, since only partial abrogation/emulation of nHZ effects is obtained with single doses of blocking antibodies/recombinant molecules, whereas the effect is fully reached after using combined doses. Nevertheless, nHZ induces a plethora of pro-inflammatory molecules [43], some of which correlate with TIMP-1 gene expression (i.e. MCP-1/CCL2, MIP-1β/CCL4, and RANTES/CCL5) [45]–[46]. Therefore, the involvement of additional cytokines and chemokines as redundant soluble mediators for nHZ-promoted TIMP-1 expression and release cannot be excluded.

We next wanted to investigate the molecular mechanisms underlying nHZ-induced upregulation of TIMP-1 and related pro-inflammatory molecules. It has been previously reported that IL-1β induces both TIMP-1 and MMP-9 in monocytes and monocytic leukemia cells [44], [47]. In a separate study using human monocytes, gene expression of TIMP-1, MMP-9 and TNF-α, required p38 MAPK and NF-κB pathway activation [48]–[49]. Interestingly, several *in vitro* and *in vivo* studies have raised the possibility that either MAPK or NF-κB routes are involved in malaria.

To date we understand very little about nHZ-dependent activation of MAPKs. However, recent emerging evidence in a murine malaria model shows that nHZ induces the activation of p38 [49] and ERK1/2 [50]–[53] MAPKs, but does not induce the activation of other signaling pathways such as the JNK-2/STAT pathway [47]–[49]. Evidence from a human malaria model shows nHZ activates p38 MAPK signaling, similar to the murine model, whilst nHZ does not appear to activate the ERK1/2 or JNK-1/2 signaling pathways in the human model [25], [31], [54]. Interestingly, a study using syncytiotrophoblast cells shows nHZ-dependent phosphorylation of ERK1/2, along with IRBC-dependent phosphorylation of JNK-1, with both events being causally related to production of pro-inflammatory molecules (TNF-α, MIP-1α/CCL3, IL-8/CXCL8) [55]–[56]. Moreover, our group recently showed intravenous injection of nHZ in malaria-free mice induces an inflammatory frame similar to that observed in experimental malaria-associated acute respiratory distress syndrome, pointing to nHZ as a prominent inflammatory virulent factor in lung pathology [26]. On the other hand, *Plasmodium falciparum* glycosylphosphatidylinositol (*Pf*GPI) promotes phosphorylation of all major MAPK routes, including p38, ERK1/2, and JNK-2/STAT-1 [57]–[58]. Inhibition of *Pf*GPI-dependent activation of MAPKs decreases inflammatory responses and enhances phagocytic clearance of IRBCs in mice infected by *Plasmodium berghei* or *chabaudi chabaudi* [59]. Future research defining the differential roles of nHZ, IRBCs, and GPI in regulating MAPKs and inflammation in malaria, both *in vitro* and *in vivo*, will be certainly welcomed.

Along with nHZ-dependent regulation of MAPKs, emerging evidence suggests malarial pigment plays a role in activating the NF-kB pathway. In murine macrophages fed with nHZ or sHZ, NF-κB activation is required to upregulate expression of inducible nitric oxide synthase [51] and production of several chemokines, including MIP-1α/CCL3, MIP-1β/CCL4, MIP-2/CXCL2, and MCP-1/CCL2 [52]. Gene expression profiling of sHZ-laden RAW 264.7 macrophage cells display altered NF-κB signal transduction, enhanced inflammatory response and a severe MMP-9/TIMP-1 imbalance in favor of ECM proteolysis [60]. NF-κB activation is also mandatory for nHZ-induced MMP-9 expression in human THP-1 monocyte cell line [61] and for nHZ-enhanced release of MMP-9, lysozyme, TNF-α and IL-1β from human adherent monocytes isolated from peripheral blood [13], [25], [31]. Consistently, in peripheral mononuclear cells of malaria patients, phospho-NF-κB p65 levels are significantly higher than in healthy controls [62], and genome wide expression profiles display NF-κB-dependent enhancement of inflammatory cytokines [63].

Results from the present work suggest that nHZ-induced secretion of TIMP-1 and related pro-inflammatory molecules from human monocytes require the involvement of either p38 MAPK- or NF-κB-dependent mechanisms. Indeed, the effects of nHZ-mediated protein release of TIMP-1, TNF-α, IL-1β and MIP-1α/CCL3 are fully abrogated by using micromolar doses of SB203580, a synthetic inhibitor of p38 MAPK pathway [34], or after treatment with quercetin, artemisinin and parthenolide - three molecules showing antimalarial properties [64]–[65] able to block NF-κB pathway at different levels (I-κBα phosphorylation and subsequent degradation [35]; NF-κB nuclear translocation [36]; and NF-κB binding to DNA [37], respectively). Interestingly, these molecules also abrogate lysozyme and MMP-9 release in the same cellular model [13], [25], [31], [54].

Collectively, the present data show phagocytosis of nHZ by human monocytes induces inflammation-mediated expression and release of TIMP-1 through p38 MAPK- and NF-κB-dependent mechanisms, suggesting that *in vivo* nHZ-fed human monocytes may be a source for the high TIMP-1 serum levels found by Dietmann and colleagues in malaria patients [21]. In this context, TIMP-1 levels are also related to disease severity. However, it remains unclear how TIMP-1 may exacerbate the clinical course in malaria patients. One major function of TIMP-1 is to counteract and modulate the numerous effects of MMP-9, which include degradation of the sub-endothelial basal lamina, modulation of the activity of several pro-inflammatory molecules, disruption of tight junctions, and impairment of haemostasis [4]–[7]; [38], all of which comprise key roles in CM. In this way it is attractive to hypothesize that increased TIMP-1 levels are actually protective rather than a risk factor in malaria prognosis, as they could contrast the potentially detrimental effects of nHZ-enhanced MMP-9. However, our results show that MMP-9/TIMP-1 stoichiometric ratios and total gelatinolytic activity measured in nHZ-fed monocyte supernatants were significantly higher than in controls, arguing against TIMP-1 ability to act as a protective factor in this context. Total gelatinolytic activity reflects the net overall activity of gelatinases (MMP-2 and -9) and their endogenous inhibitors (TIMP-2 and TIMP-1, respectively) [33], but in the present model it should be noted that the activity is due solely to the net balance between MMP-9 and TIMP-1, as unfed and nHZ-fed human adherent monocytes do not release MMP-2 and TIMP-2 proteins [41]. Therefore, these results suggest that nHZ-dependent induction of TIMP-1 expression and release is not sufficient to counterbalance nHZ-enhanced MMP-9 levels released from human monocytes.

Intriguingly, TIMP-1 could play a detrimental role through an MMP-independent mechanism [39]–[40]. MMP-independent roles for TIMP-1 in biological processes include anti-apoptotic effects of TIMP-1 in several human cells, such as Burkitt’s lymphoma [66], breast epithelial cells [67], and cardiomyocytes [68]. Recently CD63, a member of the tetraspanin family, was identified as a cell-binding partner for TIMP-1 in human mammary epithelial cells, and CD63 down-regulation with shRNA resulted in reduced TIMP-1 binding and restored cell apoptosis [69]. Interestingly, nHZ-fed monocytes do not undergo apoptosis, despite increased inflammation and functional impairment [30], [43]. CD63 is constitutively expressed by human monocytes [70]. Thus, it is intriguing to speculate that nHZ-enhanced TIMP-1 levels might prevent apoptosis of functionally impaired nHZ-fed cells through CD63-dependent mechanisms. Additionally, there are several reports describing MMP-independent abilities of TIMP-1 to inhibit cell growth and differentiation [39]–[40], [71]. These TIMP-1 properties may be crucial for nHZ-fed monocytes, since these cells have been shown not to mature to dendritic cells [72] and not to coordinate erythropoiesis [73].

In conclusion, the present work expands the available evidence on the expression of TIMPs in malaria, providing new information on the mechanisms underlying nHZ-dependent TIMP-1 increase. Future investigation is needed to ascertain whether nHZ-enhanced TIMP-1 may contribute to worsen the clinical course in malaria patients as a consequence of its MMP-independent anti-apoptotic or growth/differentiation-inhibitory properties. Further, previous evidence correlating the number of circulating nHZ-laden monocytes in patients to parasitaemia degree and malaria severity [29] seems to support such a hypothesis. More extensive research on the functional role of TIMP-1 in malaria, along with a better understanding of MMP-independent TIMP functions is necessary in order to find new tools for differential diagnosis and therapy of severe malaria.

## Supporting Information

Figure S1 **Phagocytic meals and treatments do not display cytotoxicity and do not affect viability of human adherent monocytes.** Cells were left unfed or fed with nHZ and latex for 2 h; after washing, nHZ-fed cells were incubated for 24 h alone, whereas unfed cells were incubated for 24 h with 30 ng/ml of anti-hTNF-α, anti-hIL-1β, anti-hMIP-1α/CCL3 blocking antibodies; 20 ng/ml of rhTNF-α, rhIL-1β, rhMIP-1α/CCL3; 10 µM SB203580; 15 µM quercetin; 10 µM artemisinin; and 10 µM parthenolide. Thereafter, cell supernatants and lysates were collected and LDH activity was measured by a spectrometric assay. Panel A. Cytotoxicity of phagocytic meals and treatments, expressed as percentage of (extracellular LDH activity)/(total LDH activity) ratio *versus* controls (unfed/untreated monocytes). Panel B. Viability of cells after exposure to phagocytic meals and treatments, expressed as percentage of (intracellular LDH activity)/(total LDH activity) ratio *versus* controls (unfed untreated monocytes). Data are mean values+SEM of three independent experiments. All data were evaluated for significance by ANOVA: no significant differences were found.
(TIF)

Figure S2 **Co-culturing with nHZ-fed monocytes induces unfed cells to release TIMP-1 and MMP-9.** Unfed monocytes (1×10^6^ cells/well) were plated at the bottom of the wells, with nHZ-fed human adherent monocytes (0,5×10^6^ cells/well) seeded onto the inserts. Co-cultures were incubated for 2 h before removal of the inserts. After washings, unfed monocytes were further incubated for 24 h. Non-co-cultured unfed and nHZ-cells were also used as negative and positive controls, respectively. Cell supernatants were collected and analysed for TIMP-1 and MMP-9 secretion by ELISA. Panel A. Secretion of TIMP-1 (white columns), expressed as pg/ml. Panel B. Secretion of MMP-9 (white columns) and TIMP-1 (black columns), expressed as pmol/ml. Data are mean values+SEM of three independent experiments. All data were evaluated for significance by ANOVA. Panel A: Vs non-co-cultured unfed cells (column 1) *p<0.0001; Vs non-co-cultured nHZ-fed cells (column 3) °p<0.0001. Panel B: Vs non-co-cultured unfed cells (column 1) *p<0.0001.
(TIF)
