# Supplementary figures and images for: Natural Haemozoin Induces Expression and Release of Human Monocyte Tissue Inhibitor of Metalloproteinase-1
Source: PLoS One. 2013 Aug 14;8(8):e71468. doi: 10.1371/journal.pone.0071468 (PMC3743797; doi:10.1371/journal.pone.0071468)

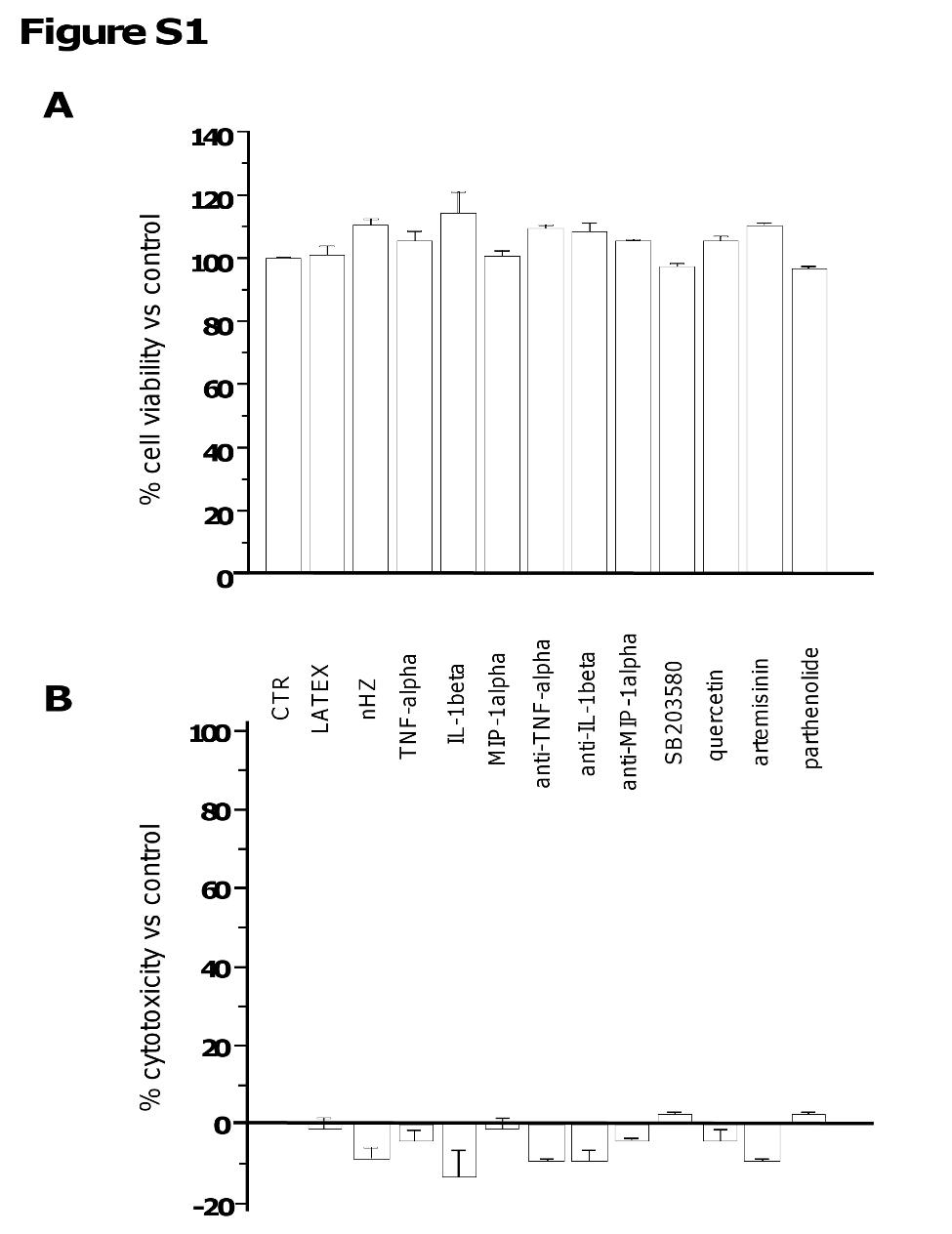

Supplement: Figure S1 — Phagocytic meals and treatments do not display cytotoxicity and do not affect viability of human adherent monocytes. Cells were left unfed or fed with nHZ and latex for 2 h; after washing, nHZ-fed cells were incubated for 24 h alone, whereas unfed cells were incubated for 24 h with 30 ng/ml of anti-hTNF-α, anti-hIL-1β, anti-hMIP-1α/CCL3 blocking antibodies; 20 ng/ml of rhTNF-α, rhIL-1β, rhMIP-1α/CCL3; 10 µM SB203580; 15 µM quercetin; 10 µM artemisinin; and 10 µM parthenolide. Thereafter, cell supernatants and lysates were collected and LDH activity was measured by a spectrometric assay. Panel A. Cytotoxicity of phagocytic meals and treatments, expressed as percentage of (extracellular LDH activity)/(total LDH activity) ratio versus controls (unfed/untreated monocytes). Panel B. Viability of cells after exposure to phagocytic meals and treatments, expressed as percentage of (intracellular LDH activity)/(total LDH activity) ratio versus controls (unfed untreated monocytes). Data are mean values+SEM of three independent experiments. All data were evaluated for significance by ANOVA: no significant differences were found. (TIF) [file pone.0071468.s001.tif]

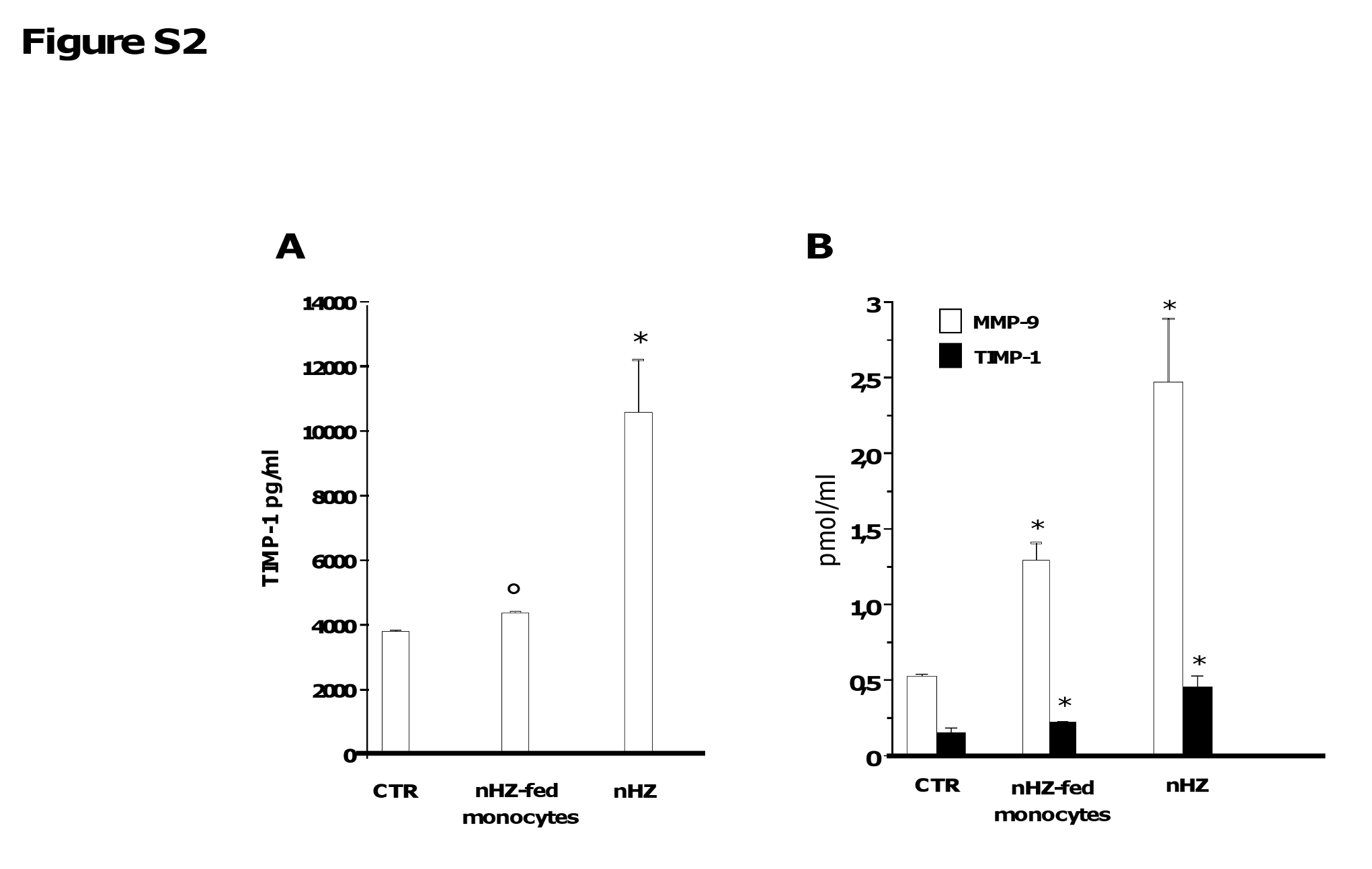

Supplement: Figure S2 — Co-culturing with nHZ-fed monocytes induces unfed cells to release TIMP-1 and MMP-9. Unfed monocytes (1×10^6^ cells/well) were plated at the bottom of the wells, with nHZ-fed human adherent monocytes (0,5×10^6^ cells/well) seeded onto the inserts. Co-cultures were incubated for 2 h before removal of the inserts. After washings, unfed monocytes were further incubated for 24 h. Non-co-cultured unfed and nHZ-cells were also used as negative and positive controls, respectively. Cell supernatants were collected and analysed for TIMP-1 and MMP-9 secretion by ELISA. Panel A. Secretion of TIMP-1 (white columns), expressed as pg/ml. Panel B. Secretion of MMP-9 (white columns) and TIMP-1 (black columns), expressed as pmol/ml. Data are mean values+SEM of three independent experiments. All data were evaluated for significance by ANOVA. Panel A: Vs non-co-cultured unfed cells (column 1) *p<0.0001; Vs non-co-cultured nHZ-fed cells (column 3) °p<0.0001. Panel B: Vs non-co-cultured unfed cells (column 1) *p<0.0001. (TIF) [file pone.0071468.s002.tif]
